# Supplementary material for: Single-cell transcriptomic profiling of the whole colony of Botrylloides diegensis: insights into tissue specialization and blastogenesis
Source: Development. 2025 Jun 17;152(20):dev204265. doi: 10.1242/dev.204265 (PMC12212465; doi:10.1242/dev.204265)
Supplement: Supplementary information [file develop-152-204265-s1.pdf]

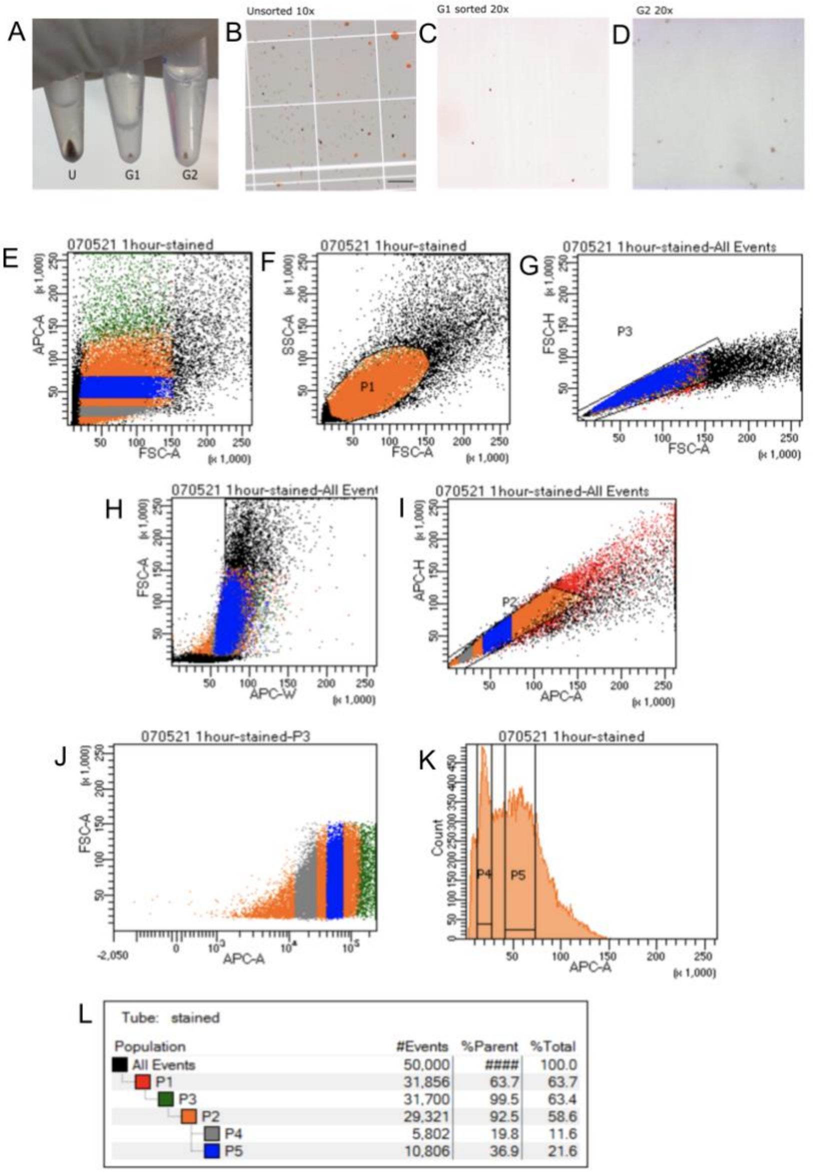

Fig. S1. ACME dissociation and single-cell sorting from mature *B. diegensis* colonies using FACS. A. The ACME cells were fixed and dissociated. A pellet was visible for the unsorted (U), and sorted G1 and G2 cells. **B.** Single-cells are apparent and intact after ACME fixation observed under fluorescent and bright field microscopy. **C.** G1 cells are present and fluorescent after sorting. **D.** G2 cells were sorted and examined under a fluorescence and bright field microscope. **E.** All events are shown after gating 50,000 single cells stained with DRAQ5. **F.** First gate selection was performed for P1 (population 1) cells based on their ideal area (FSC-A) vs. height (APC-A) signal, because higher and lower ratios are indicative of aggregates and debris, respectively. **G.** Selection of singlets with better height and size ratios within P1. **H.** Width (APC-W) vs. area of the selected P1 and P3 singlets are plotted. **I.** P2 cells were determined based on the most optimal area vs. height ratios. **J.** The area of cells correlated with their fluorescent signals. **K.** Selection of G1 (P4) and G2 (P5) cells based on their area from the P2 cells. **L.** Singlet counts of five populations and percentages of those selected from parent cell populations.

Development • Supplementary information

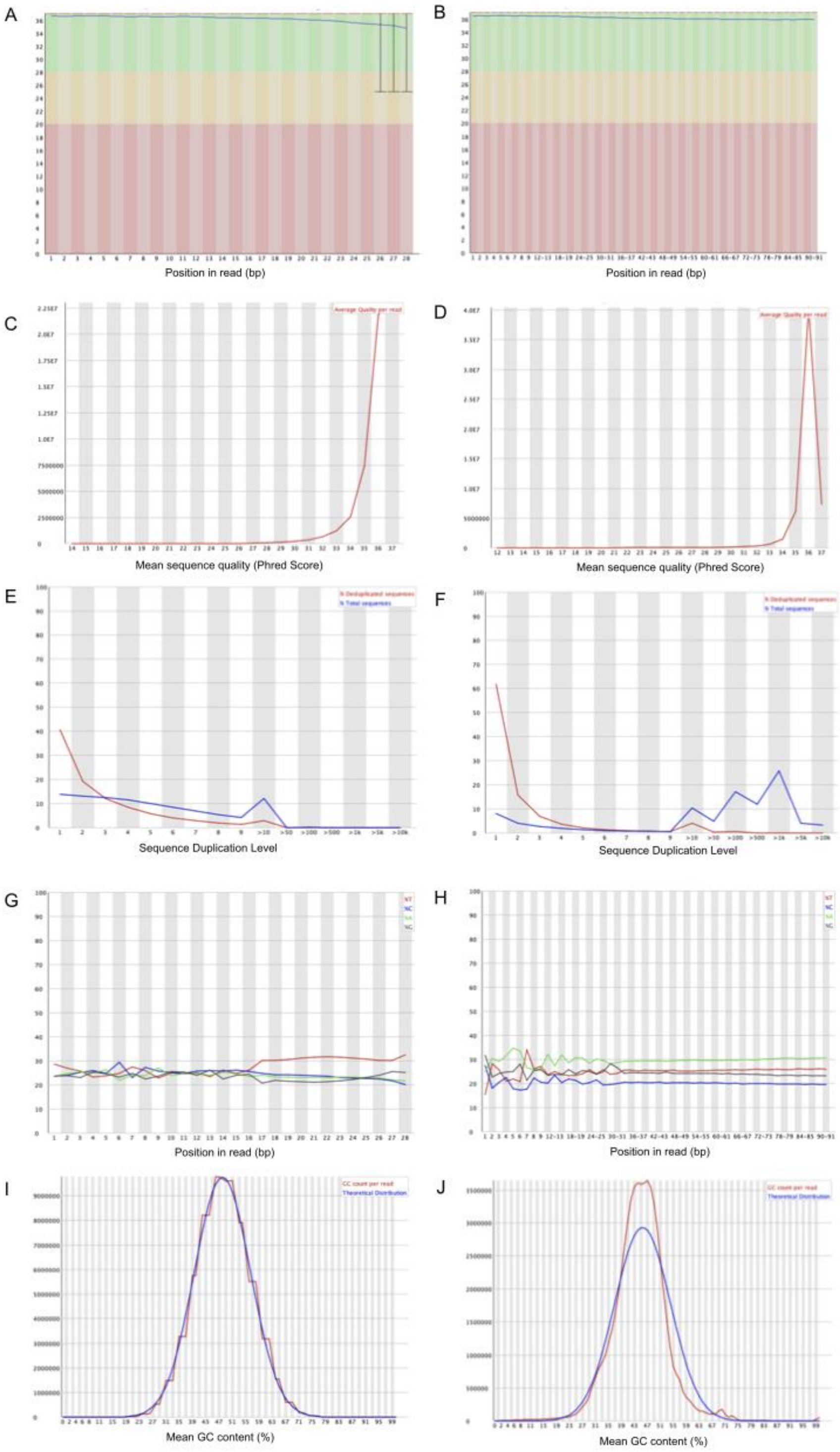

Fig. S2. FastQC quality checks were performed on the library (whole colony). Quality statistics of paired-end reads are shown for forward reads in A-C-E-G-I and reverse reads in B-D-F-H-J. **A.** Per base quality results showed forward reads with an average size of 28 bp. **B.** Reverse reads per base sequence quality were good, with an average length of 91 bp. **C.** Per-sequence quality scores are shown for forward reads. **D.** Per sequence quality scores are shown for reverse reads. **E.** Duplication of forward sequences was higher than estimated. **F.** Sequence duplication levels for reverse reads. **G.** Per base sequence content for the forward reads is shown. **H.** The sequence content of the reverse reads was plotted. **I.** Normal distribution of GC content for forward reads. **J.** Normal distribution of GC content for reverse reads.

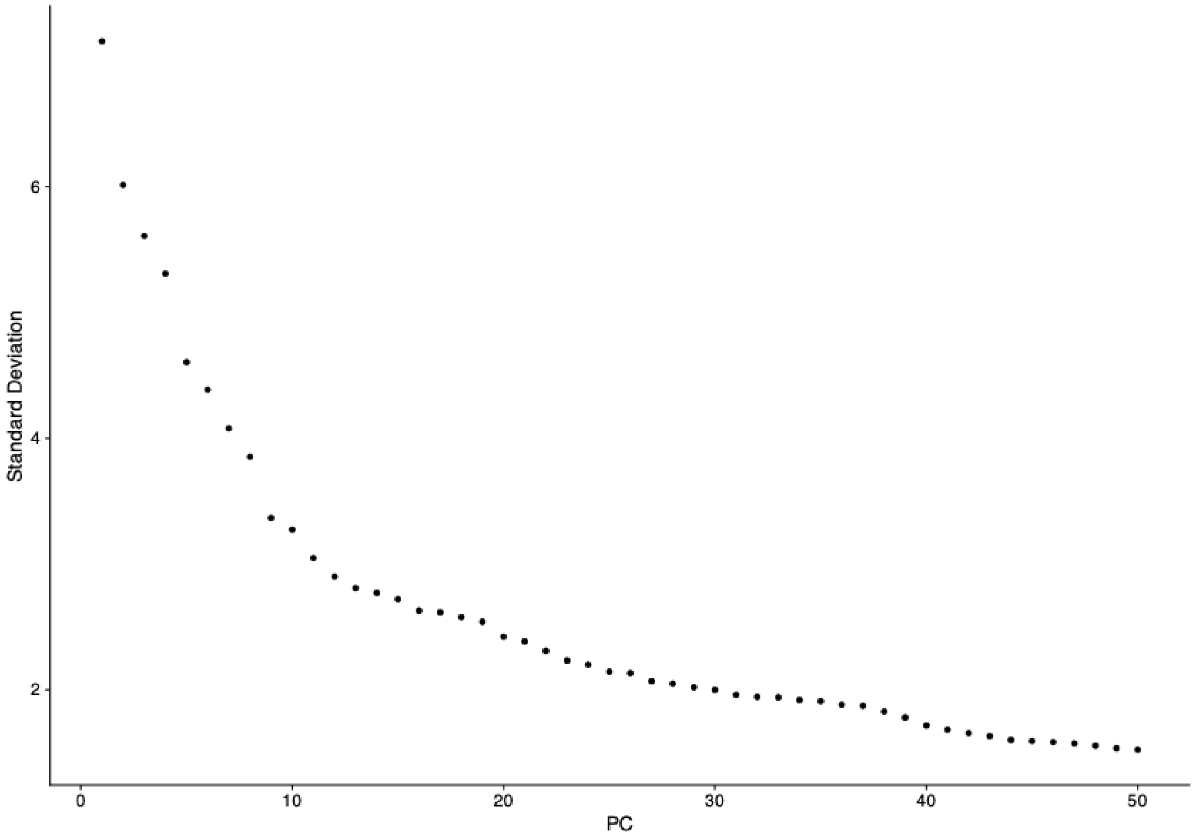

Fig. S3. Elbow Plot Showing the Standard Deviation of Principal Components

The plot displays the standard deviation associated with each principal component (PC).

Development • Supplementary information

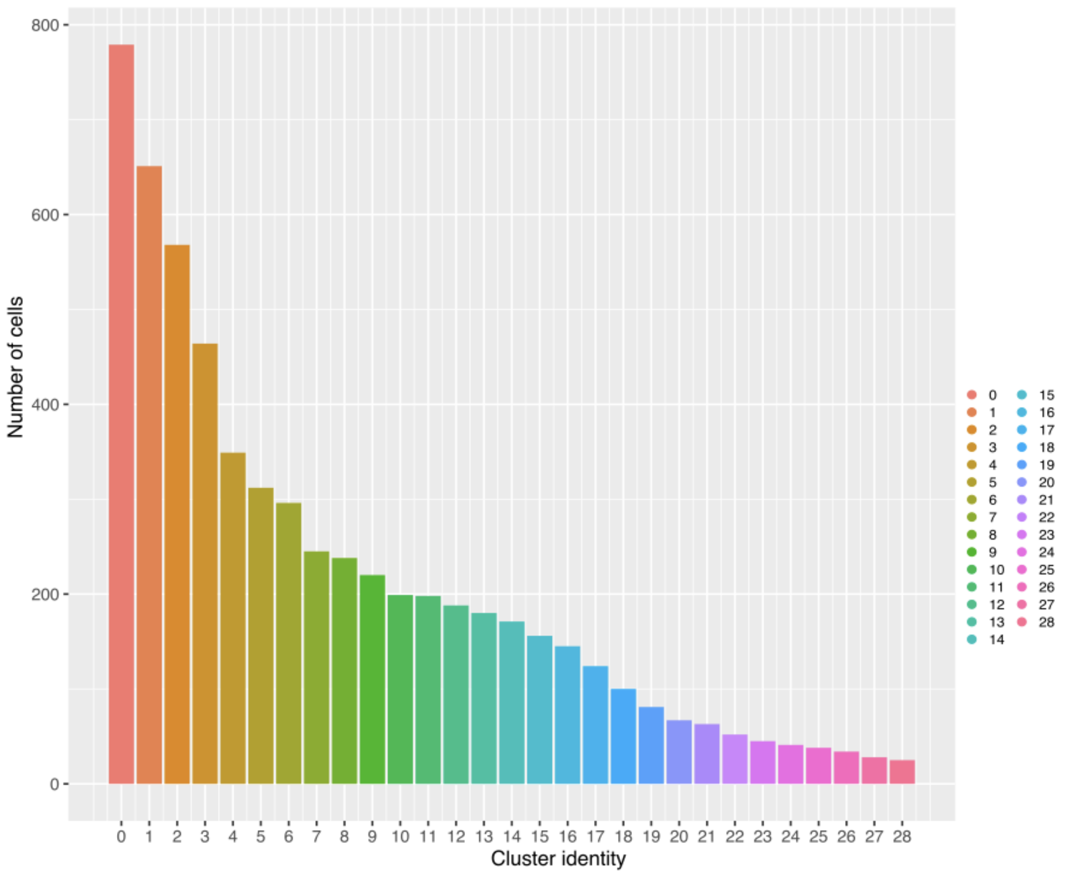

Fig. S4. The number of cells in each single-cell cluster of *B. diegensis.* Cluster 0 had the highest cell count, and the lowest was Cluster 28. The color legend reflects the cluster identities.

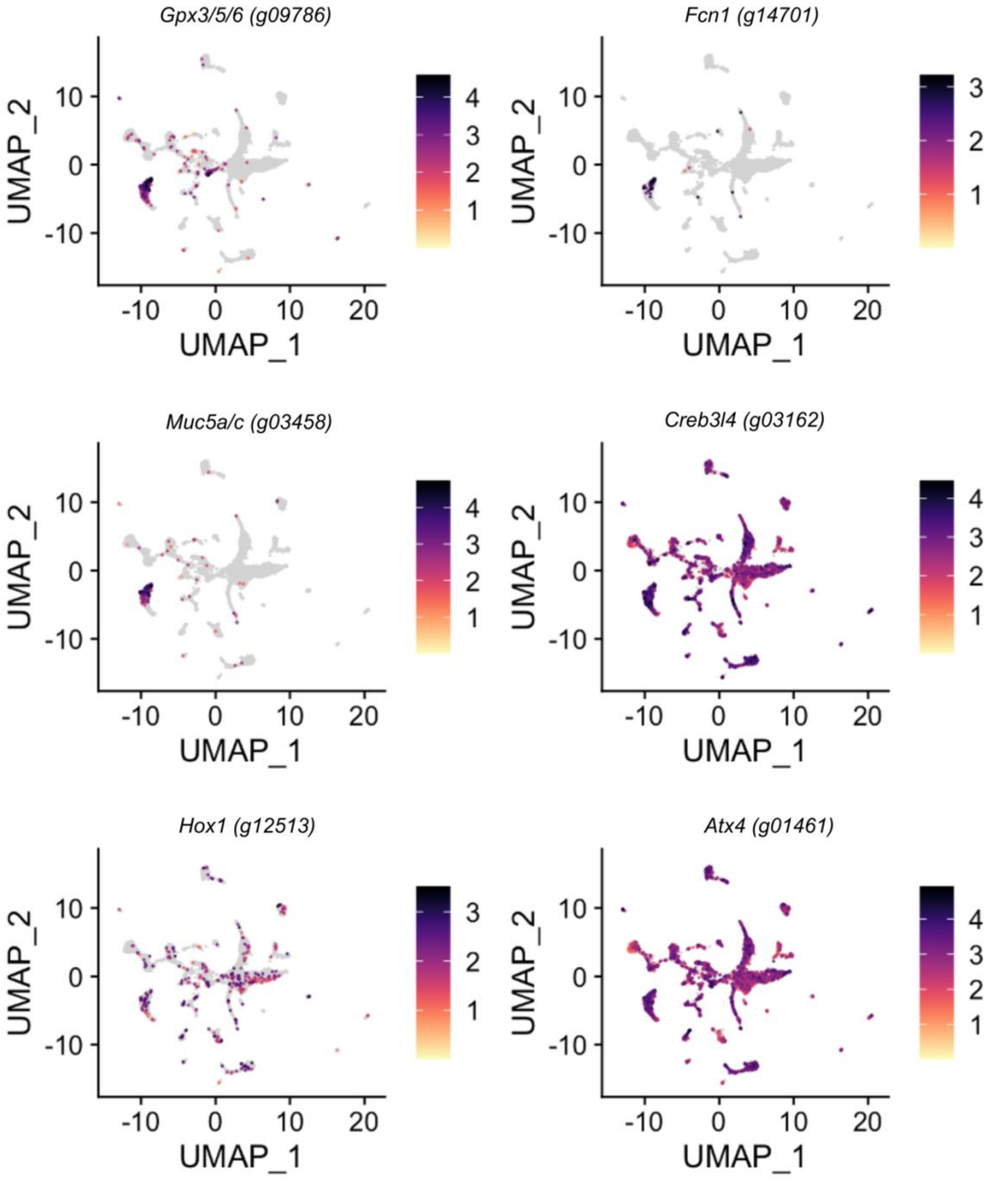

Fig. S5. UMAP featureplots for endostyle-associated genes (File S3). Feature plots for representative endostyle markers from the literature.

Development • Supplementary information

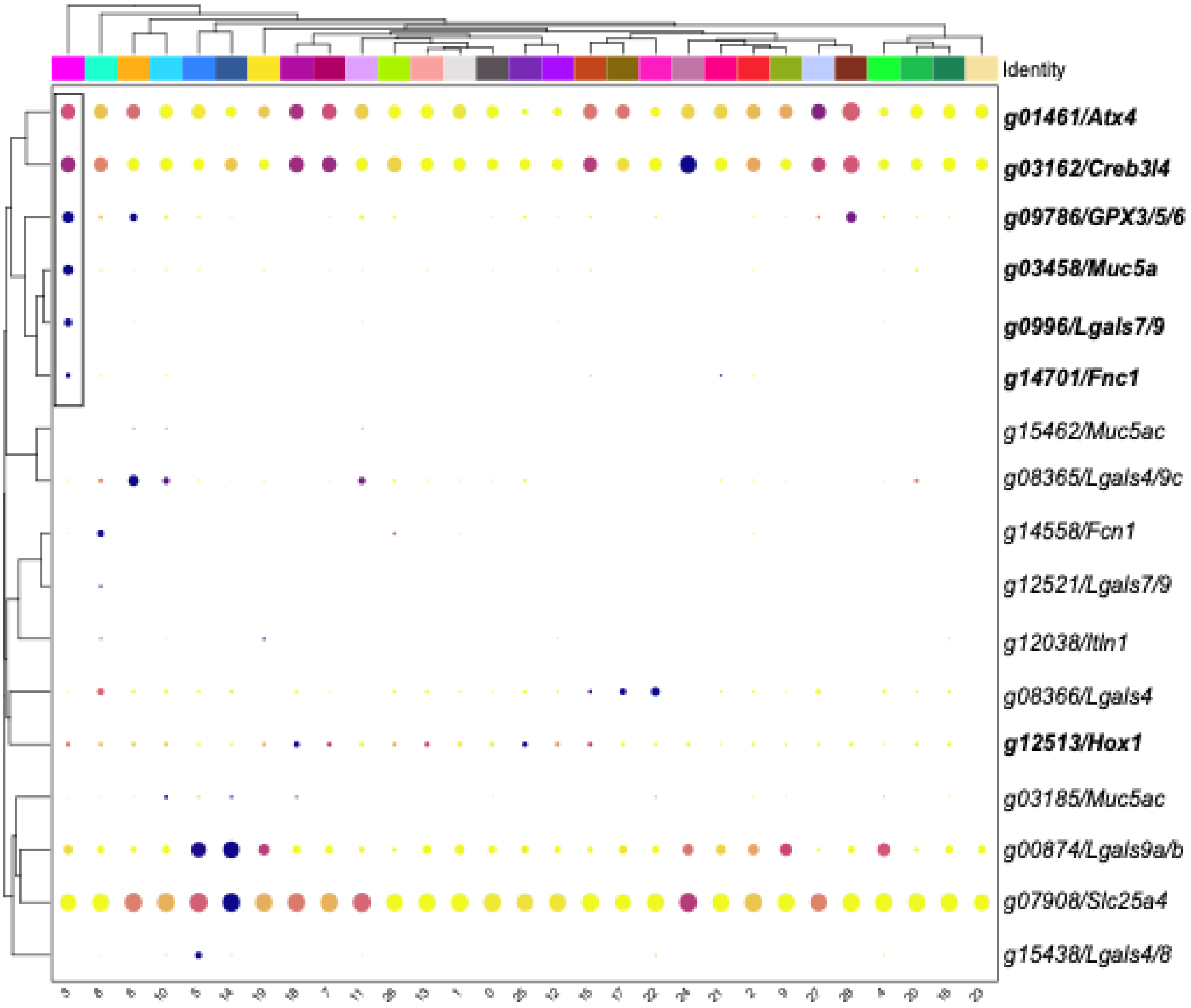

Fig. S6. Expression of candidate genes associated with the endostyle across all clusters. A clustered dot plot showing the expression patterns of seven candidate genes linked to the endostyle in other ascidians across all clusters identified in the dataset. The size of each dot represents the proportion of cells in each cluster expressing the gene, while the color intensity indicates the average expression level. Six of the seven genes exhibit strong enrichment in Cluster 3 (boxed), consistent with its predicted identity as the endostyle. The remaining gene shows a broader expression pattern across multiple clusters, including Cluster 3.

Development • Supplementary information

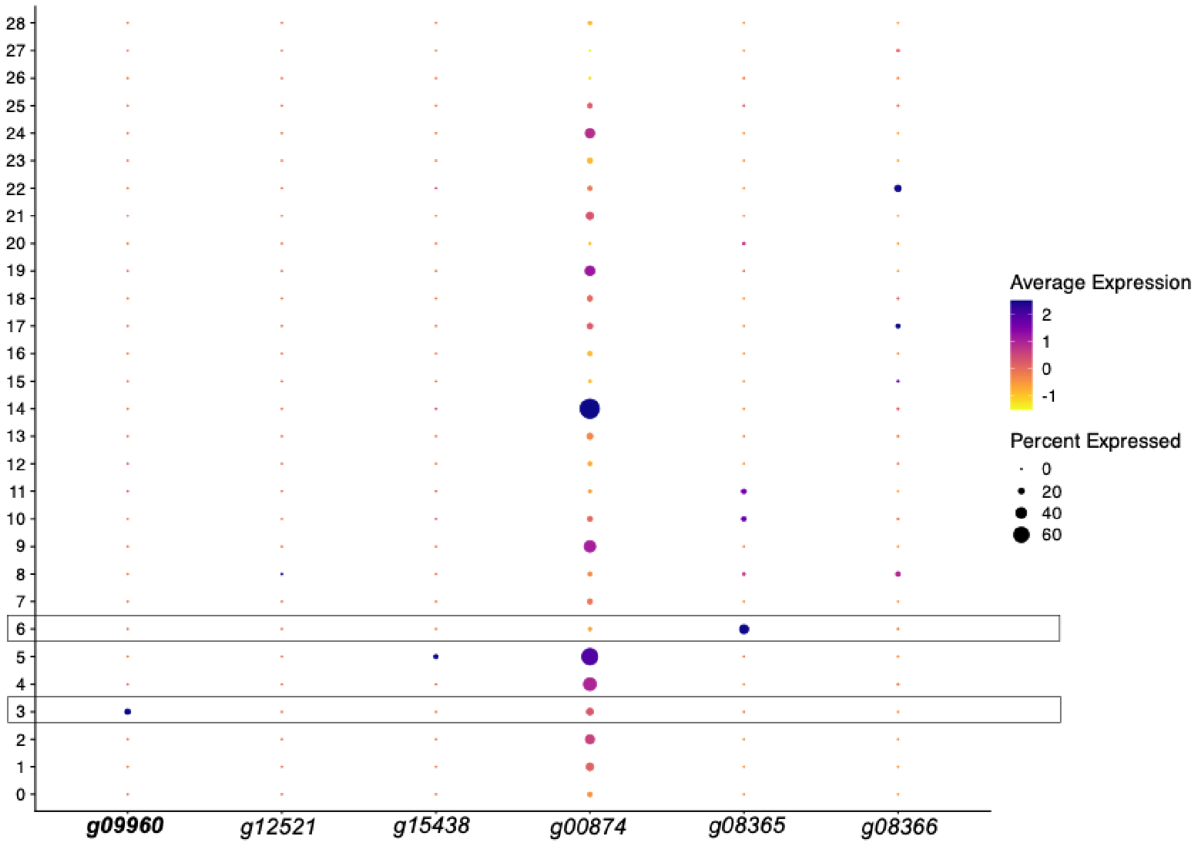

Fig. S7. Dot expression plot for five candidate L-galectin-type genes. Boxed are the clusters of interest.

Development • Supplementary information

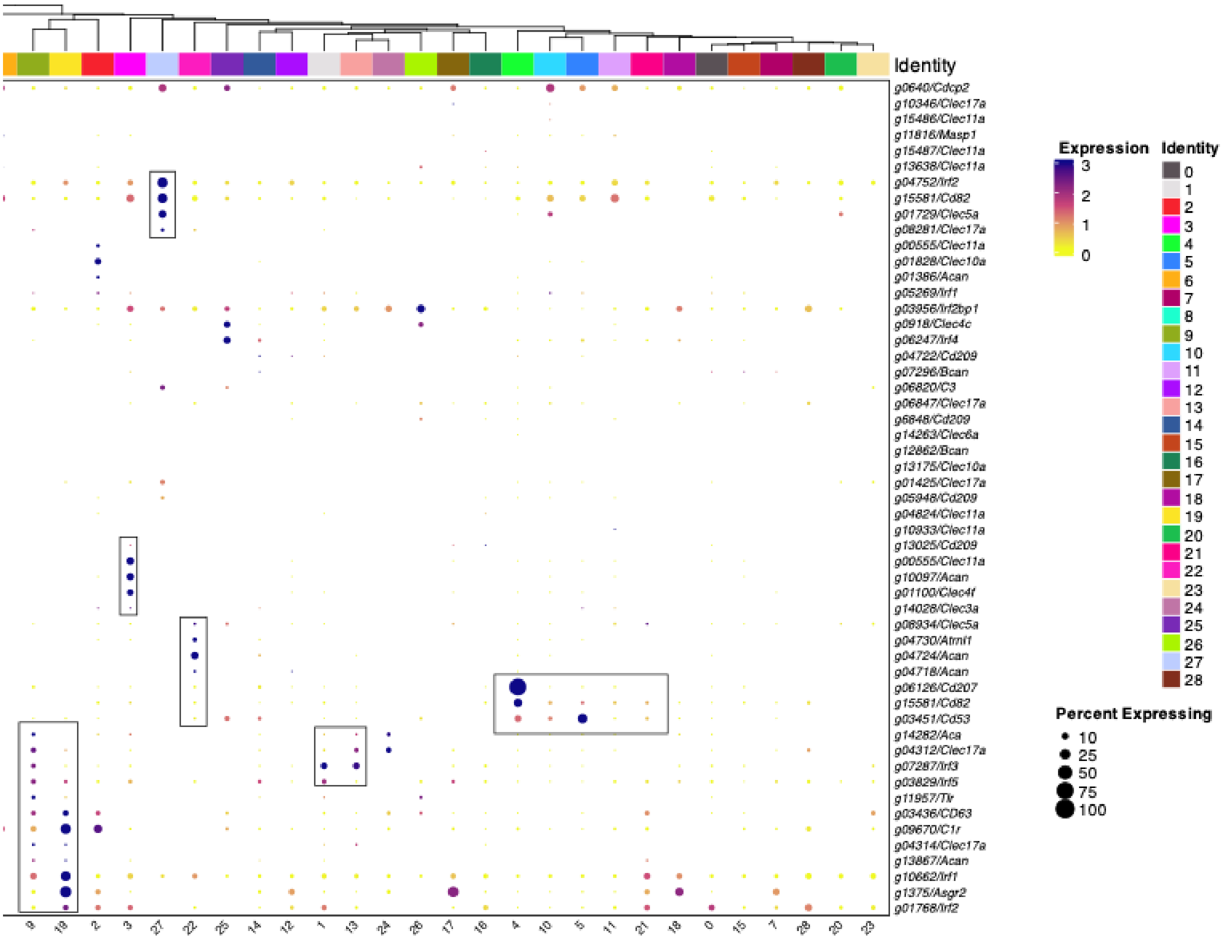

Fig. S8. Expression profiles of immune-related genes across clusters. Dot plot illustrating the expression levels and the percentage of cells expressing key immune-related genes across identified clusters in the dataset. The x-axis represents the clusters, and the y-axis lists the gene symbols, including immune-associated genes such as *Clec* family members, *Cd209*, *C3*, *Tlr*, and *Irf* genes. Dot size indicates the percentage of cells within each cluster expressing the gene, and color intensity represents the average expression level. Notable patterns indicated by boxes include cluster-specific enrichment of *Clec* genes in Cluster 8, consistent with its identification as a phagocytic cell cluster, and the grouping of *Tlr*, *Irf*, and complement-related genes in Clusters 9 and 19, potentially involved in immune regulation.

Cluster 14

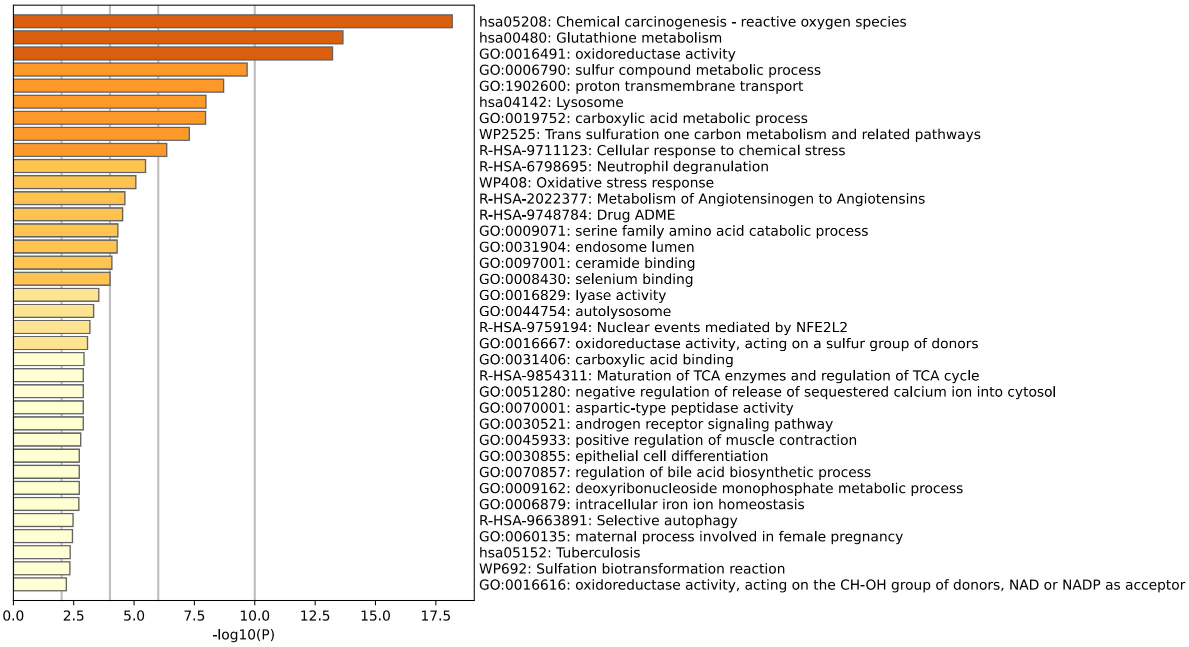

Cluster 4

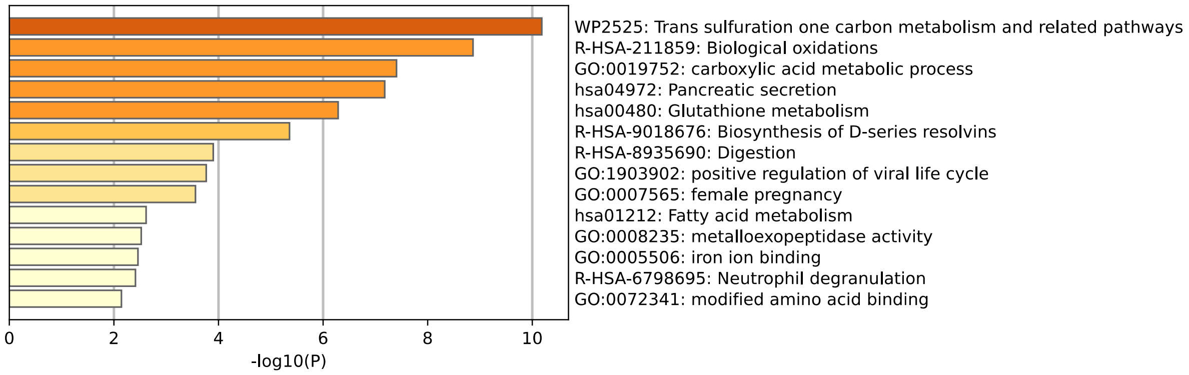

Fig. S9. Clusters 4 and 14. The bar plots for Clusters 4 and 14 highlight the enriched biological processes and pathways associated with the top marker genes for these clusters. The x-axis represents -log10(P) values, indicating the statistical significance of each enrichment term. The longer the bar, the more significant is the enrichment.

Development • Supplementary information

Cluster 10

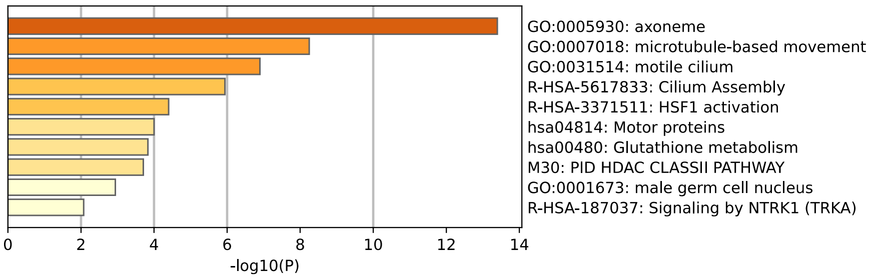

Cluster 11

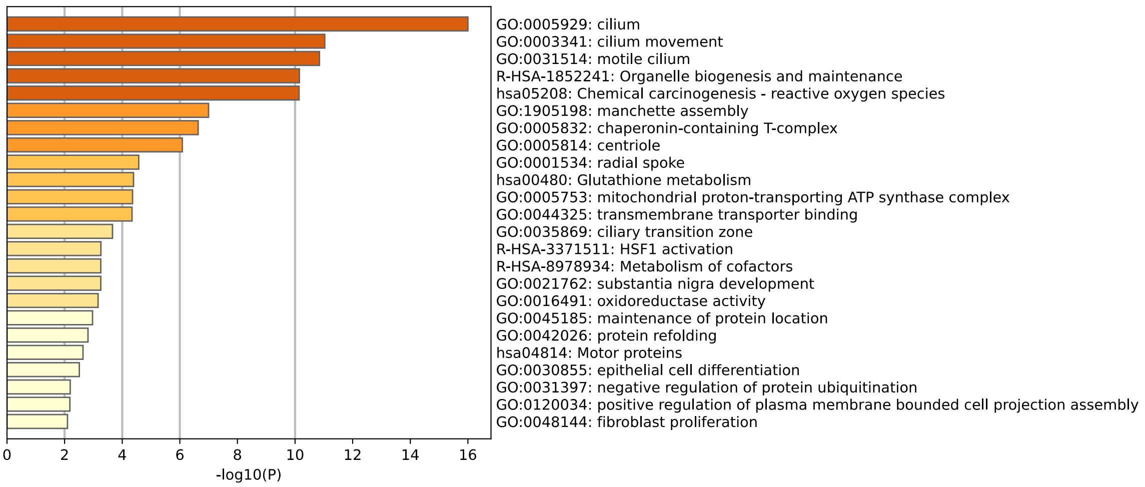

Fig. S10. Clusters 10 and 11. The bar plots for Clusters 10 and 11 highlight the enriched biological processes and pathways associated with the top marker genes for these clusters. The x-axis represents -log10(P) values, indicating the statistical significance of each enrichment term. The longer the bar, the more significant is the enrichment.

Development • Supplementary information

Cluster 0

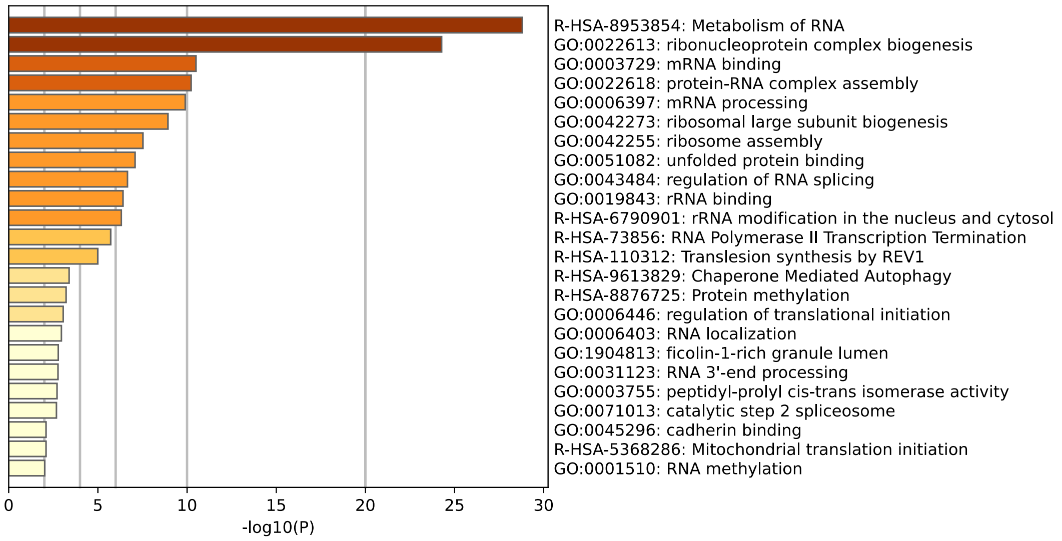

Cluster 2

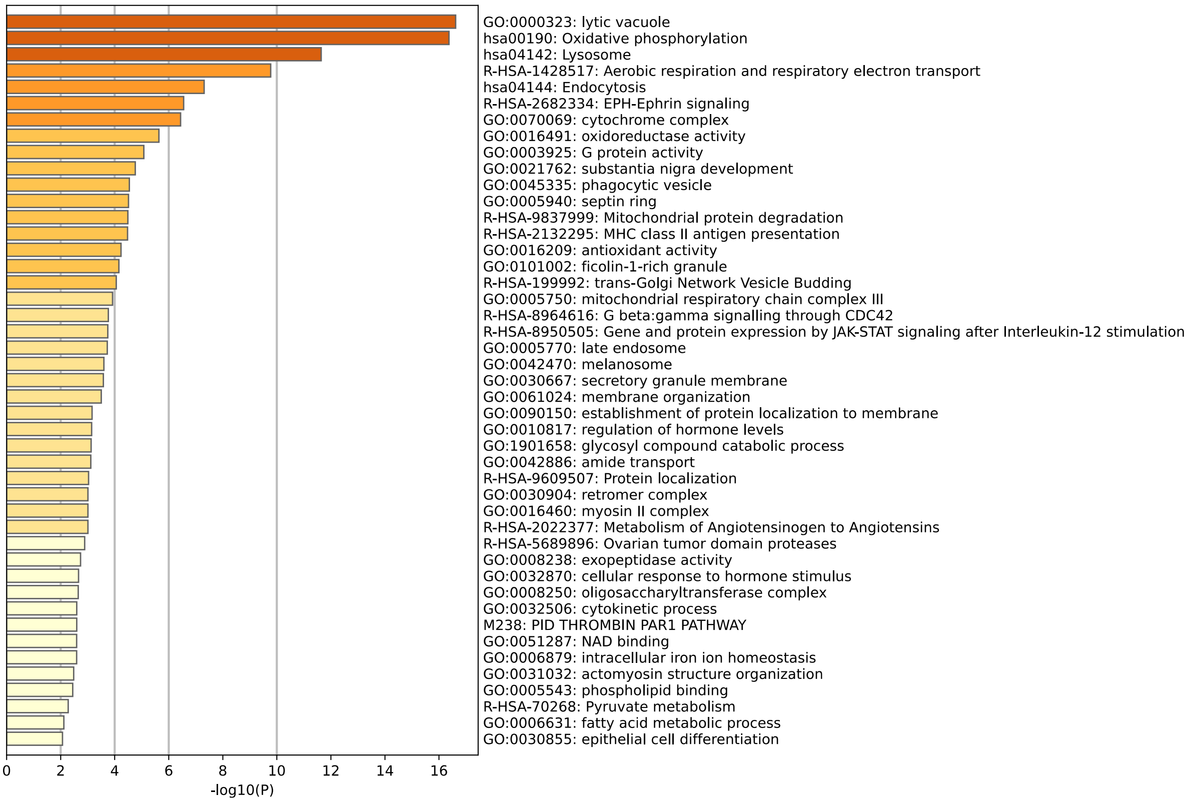

Fig. S11. Clusters 0 and 2. The bar plots for Clusters 0 and 2 highlight the enriched biological processes and pathways associated with the top marker genes for these clusters. The x-axis represents -log10(P) values, indicating the statistical significance of each enrichment term. The longer the bar, the more significant is the enrichment.

Development • Supplementary information

Cluster 3

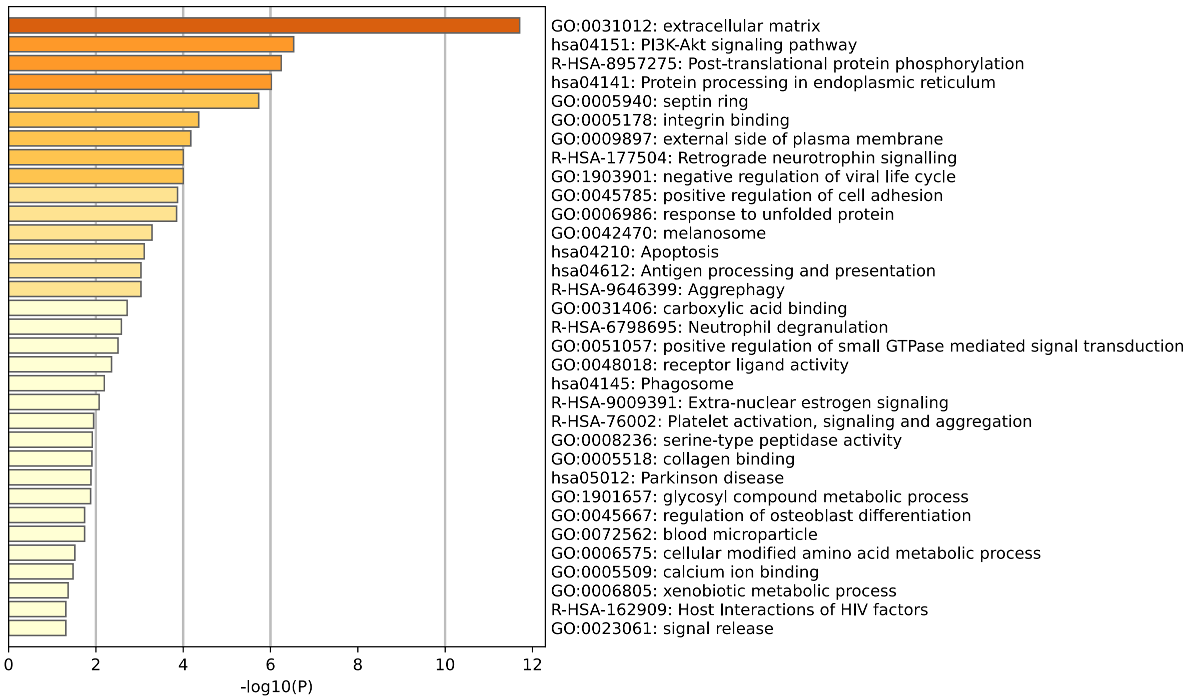

Cluster 6

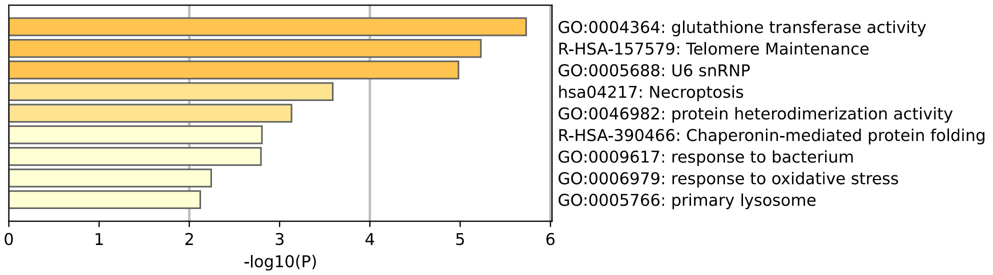

Fig. S12. Clusters 3 and 6. The bar plots for Clusters 3 and 6 highlight the enriched biological processes and pathways associated with the top marker genes for these clusters. The x-axis represents -log10(P) values, indicating the statistical significance of each enrichment term. The longer the bar, the more significant is the enrichment.

Development • Supplementary information

Cluster 9

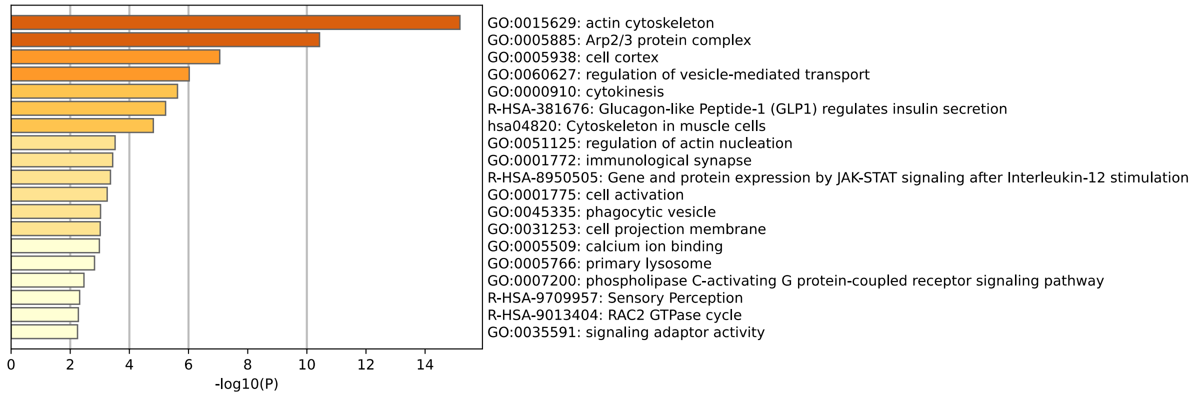

Cluster 12

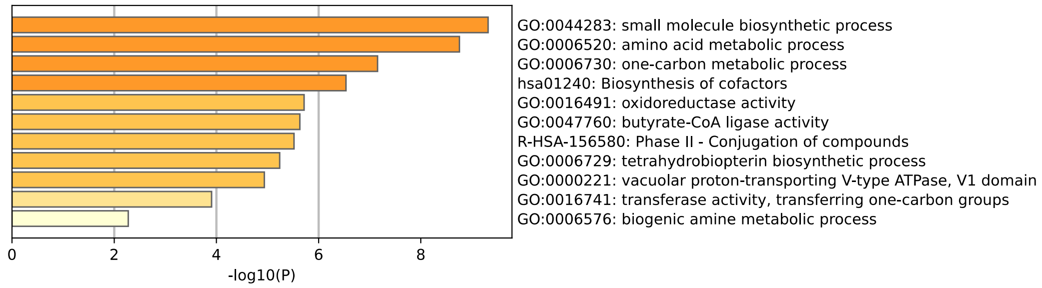

Cluster 19

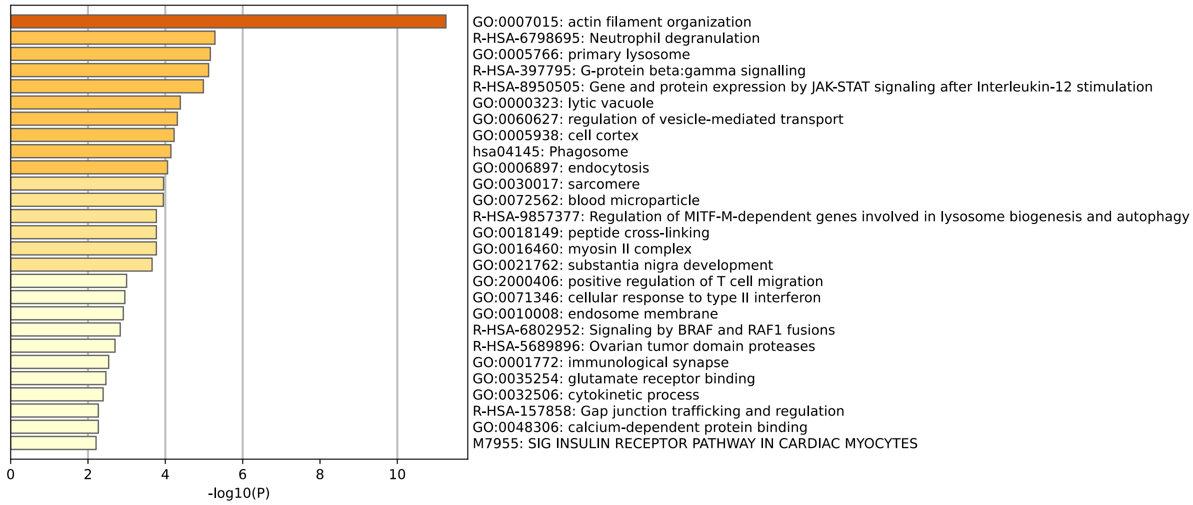

Fig. S13. Cluster 9, 19 and 12 cells.

The bar plot for Clusters 9, 19, and 12 illustrates the enriched biological processes and pathways from the top marker genes for these clusters. The x-axis represents the -log10(P) values, reflecting the statistical significance of each enrichment term. The length of each bar indicates the significance level.

Development • Supplementary information

Cluster 15

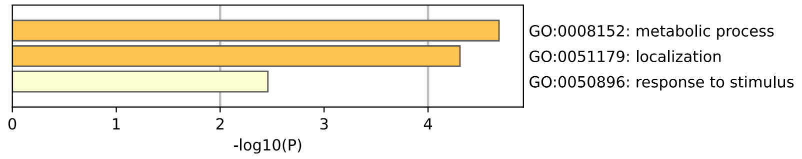

Cluster 7

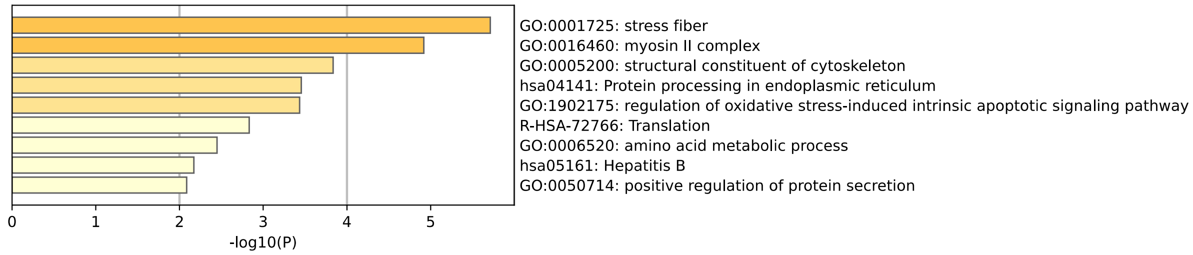

Cluster 17

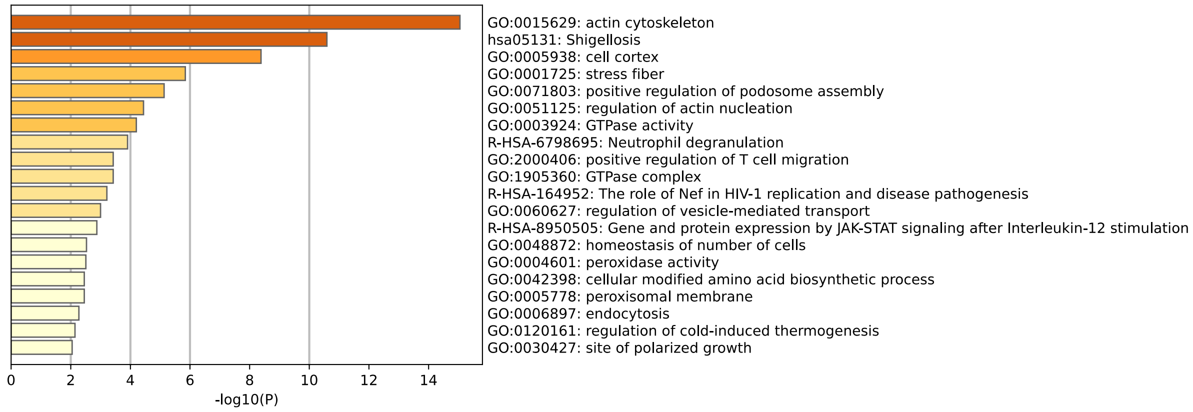

Fig. S14. Functional annotations of Clusters 15, 7 and 17.

The bar plots for Clusters 15, 7, and 17 present the enriched biological processes and pathways identified from the top marker genes for these clusters. The x-axis shows the -log10(P) values, which measure the statistical significance of each enrichment term. The longer the bar, the more significant is the enrichment.

Development • Supplementary information

Cluster 25

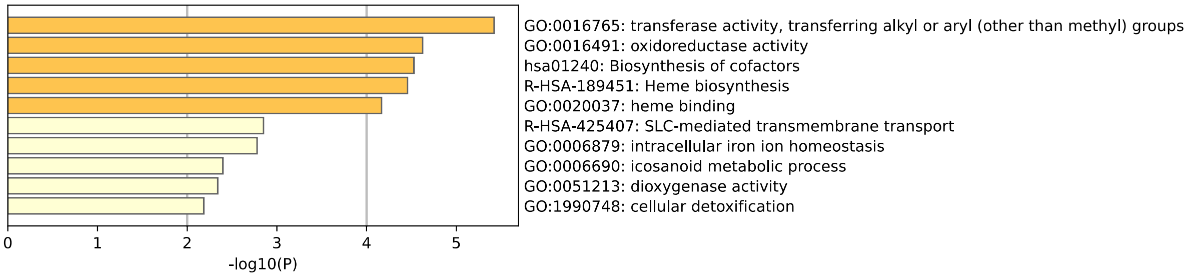

Cluster 13

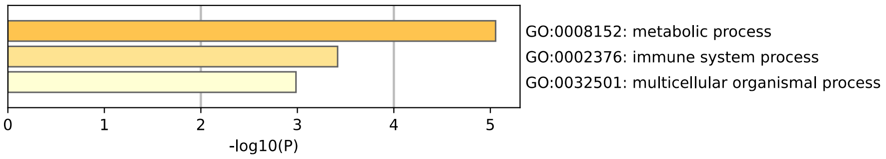

Cluster 27

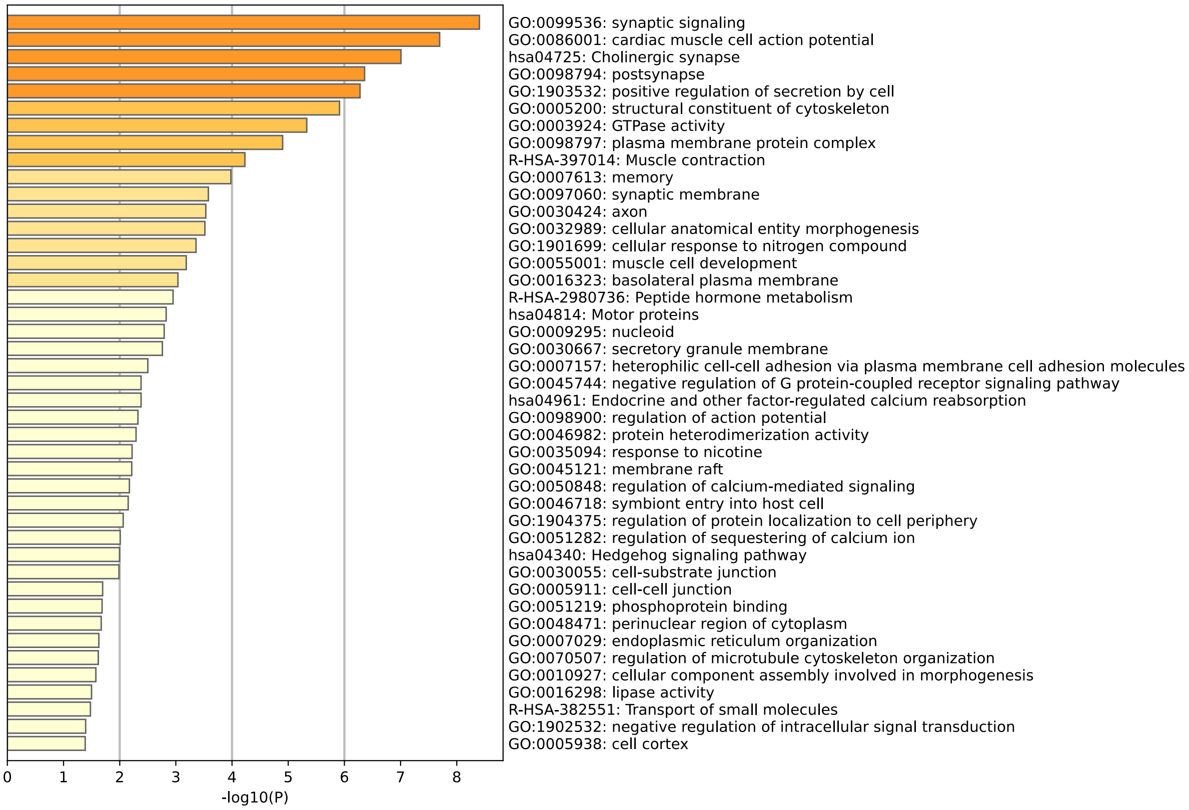

Fig. S15. Functional annotations of clusters 25, 27 and 13

The bar plot for Clusters 25, 27 and 13 highlights the enriched biological processes and pathways associated with the top marker genes for these clusters. The x-axis represents -log10(P) values, indicating the statistical significance of each enrichment term.

Development • Supplementary information

Cluster 8

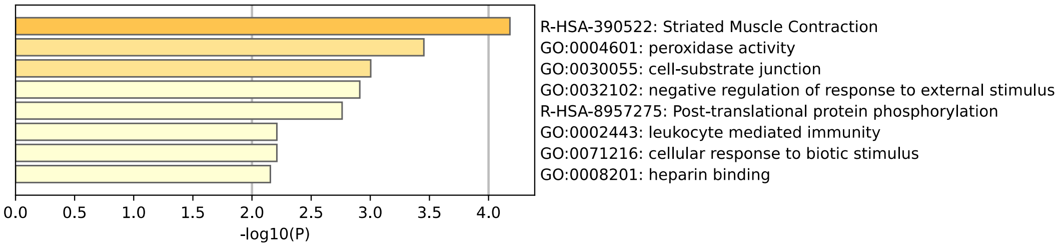

Cluster 24

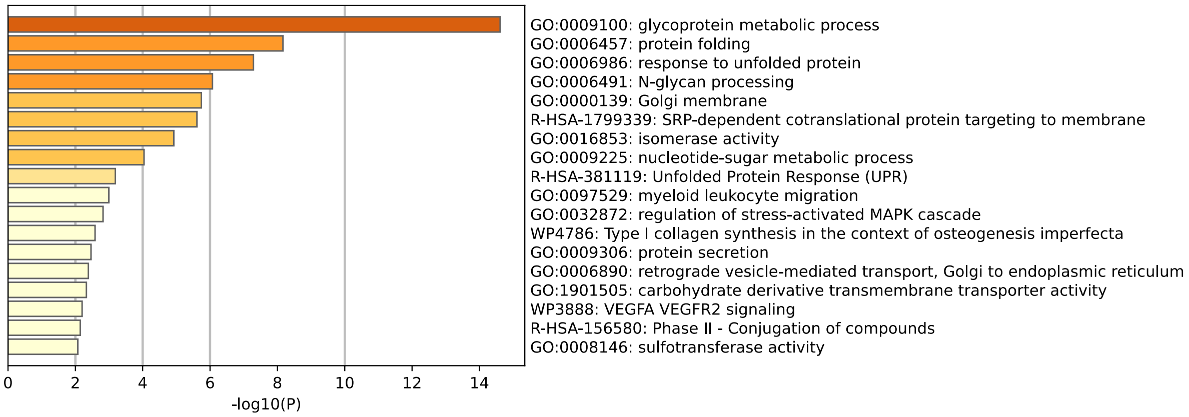

Fig. S16. Functional annotations of Clusters 8 and 24. The bar plot for Clusters 8 and 24 highlights the enriched biological processes and pathways associated with the top marker genes for these clusters. The x-axis represents -log10(P) values, indicating the statistical significance of each enrichment term.

Development • Supplementary information

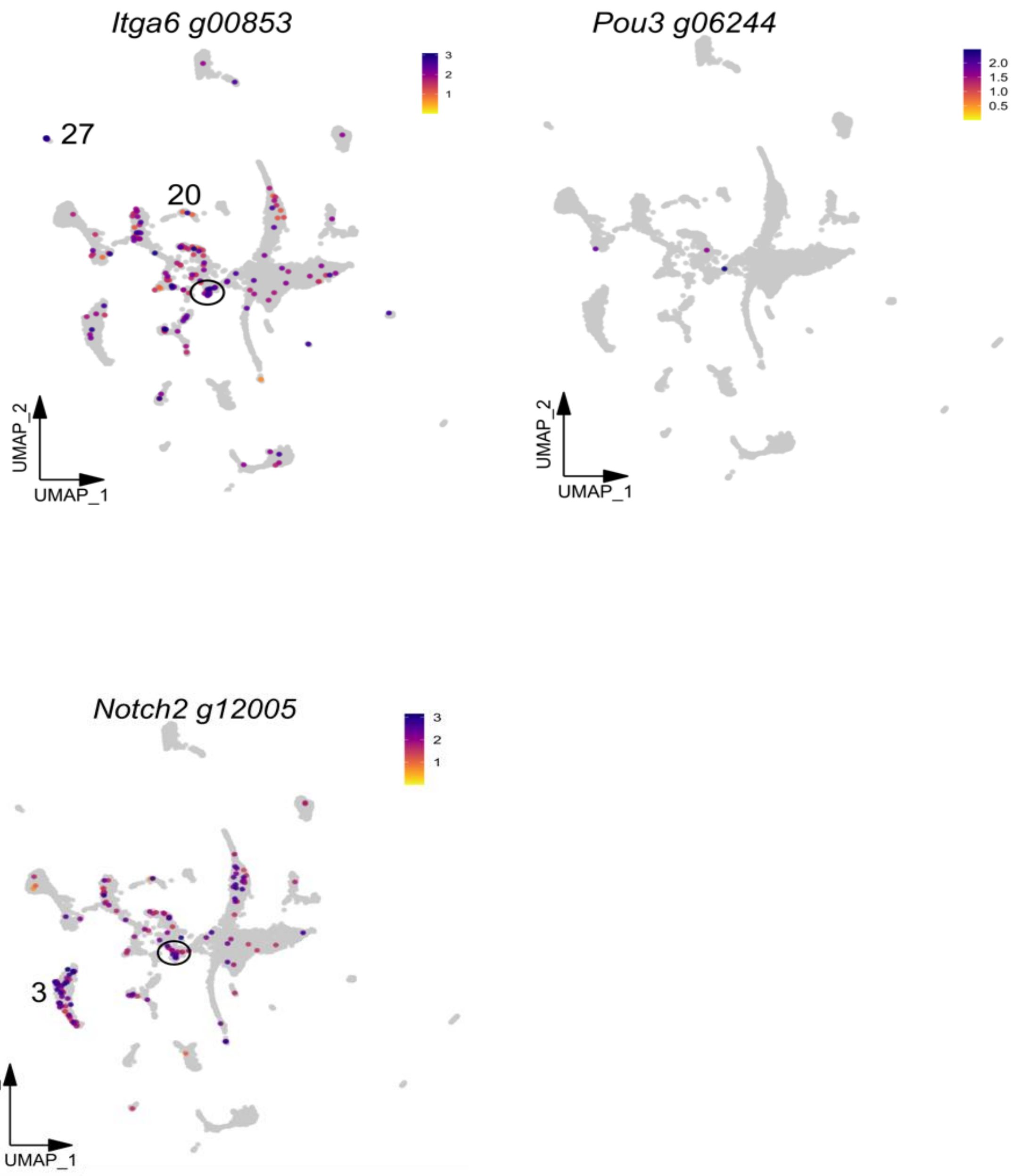

Fig. S17. Feature plots of genes previously linked to stemness in *Botrylloides* sp. Circled cells are located within Cluster 6 are of interest.

Development • Supplementary information

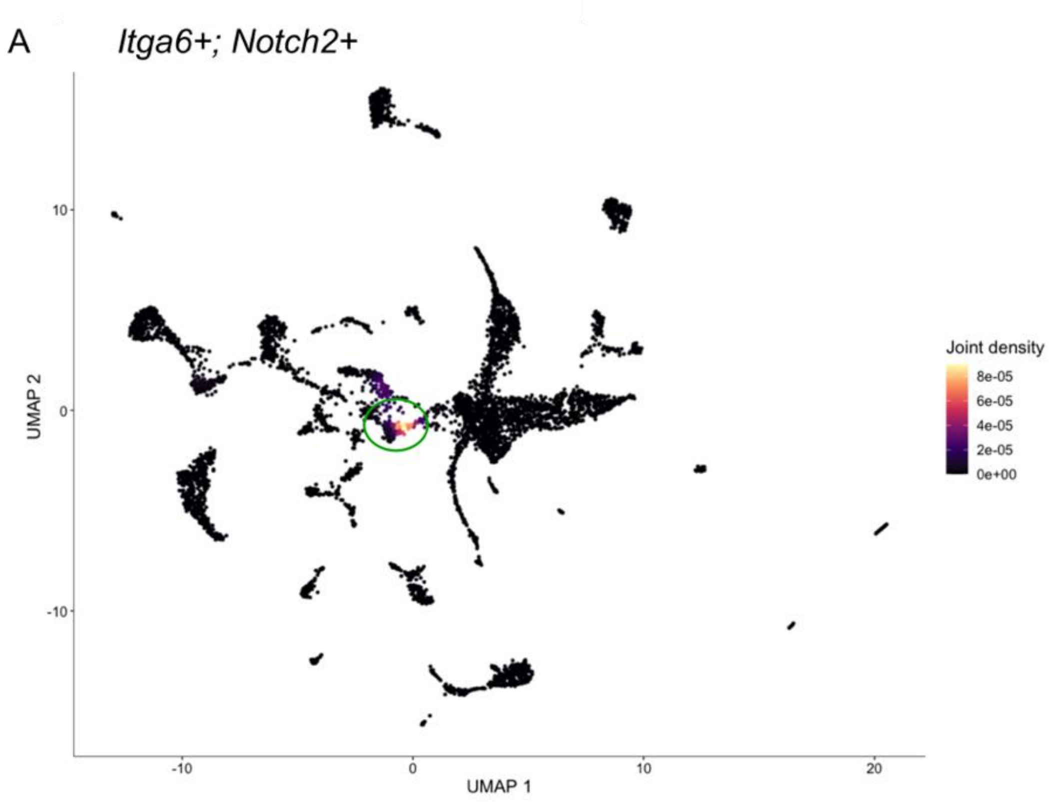

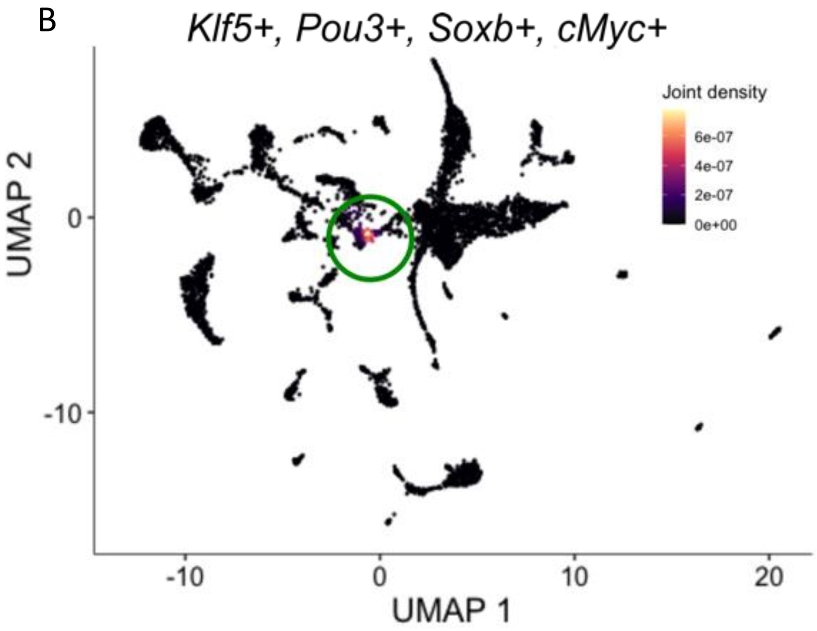

Fig. S18. Feature joint density plots. **A**. Genes linked to stem cell function. **B**. Yamanaka factors. Circled cells are located within Cluster 6, which are of interest.

Development • Supplementary information

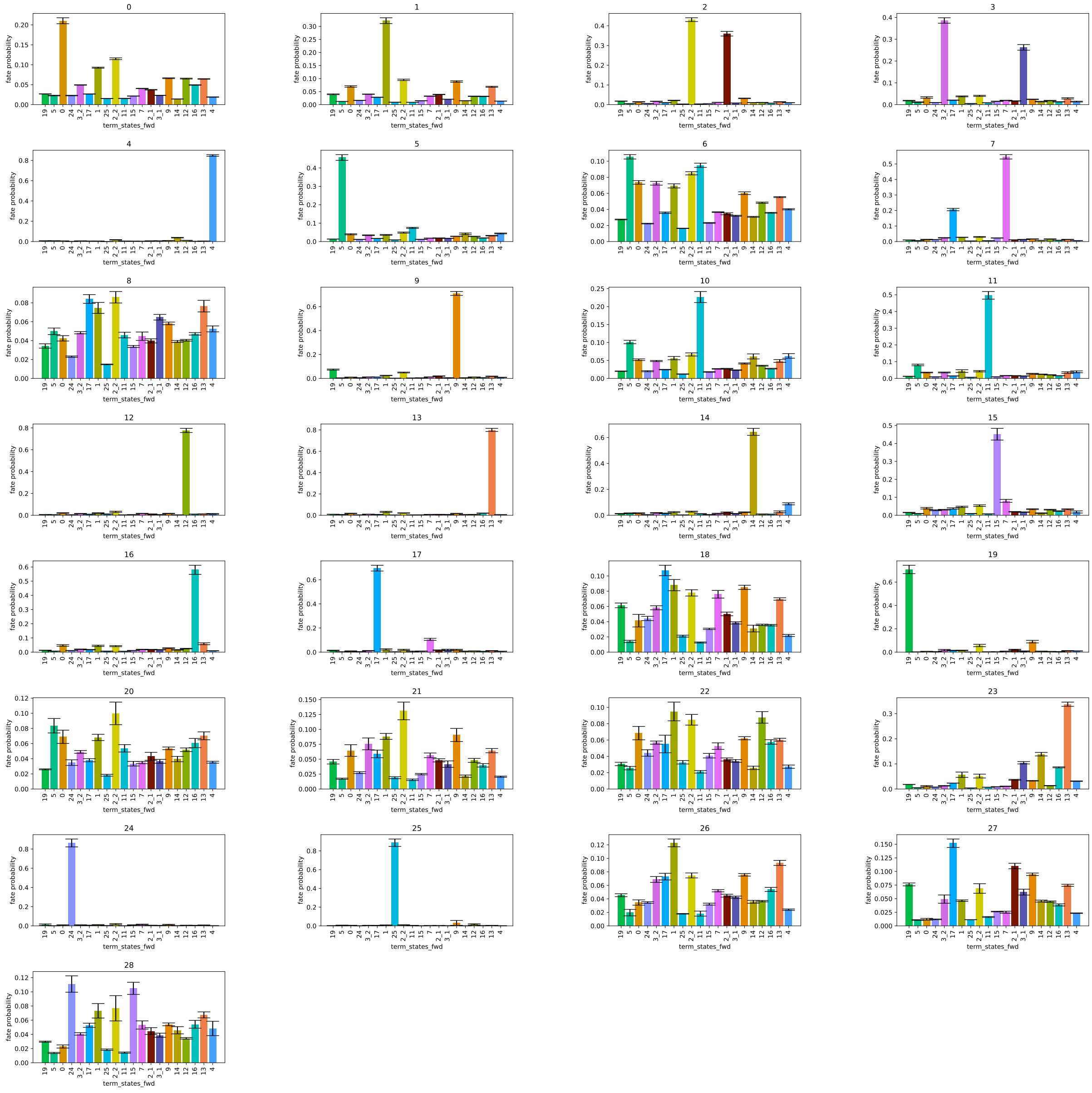

Fig. S19. CellRank cluster terminal fate probabilities. The terminal fate probabilities for each cluster are shown, as determined by CellRank analysis. Each bar plot represents the probability distribution of the terminal states of the cells within a specific cluster. The x-axis of each bar plot denotes the various terminal fates, while the y-axis represents the probability of cells within each cluster obtaining each fate.

Development • Supplementary information

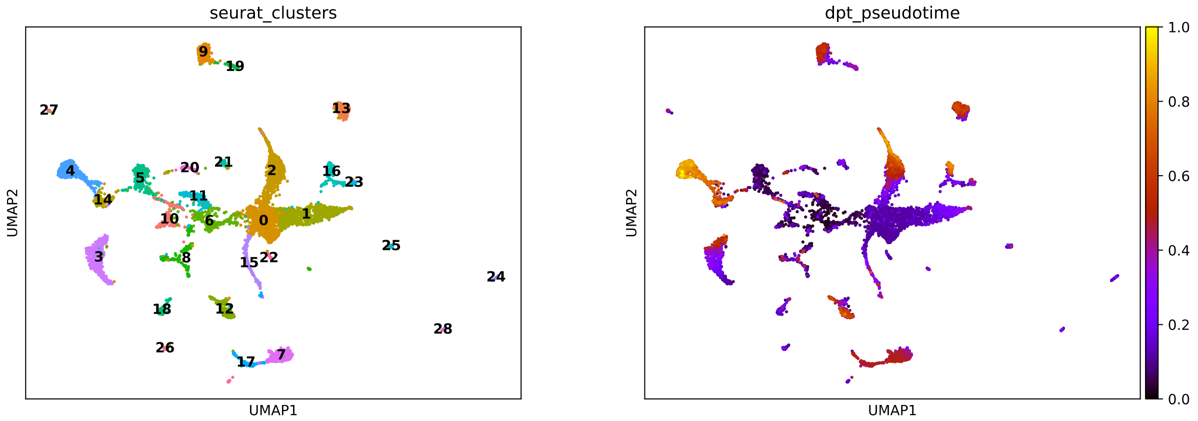

Fig. S20. UMAPs for diffusion pseudotime (dpt) and Seurat clusters. Plots generated using CellRank (v. 2.0). The higher values (dpt_pseudotime) are later in the differentiation trajectory.

Development • Supplementary information

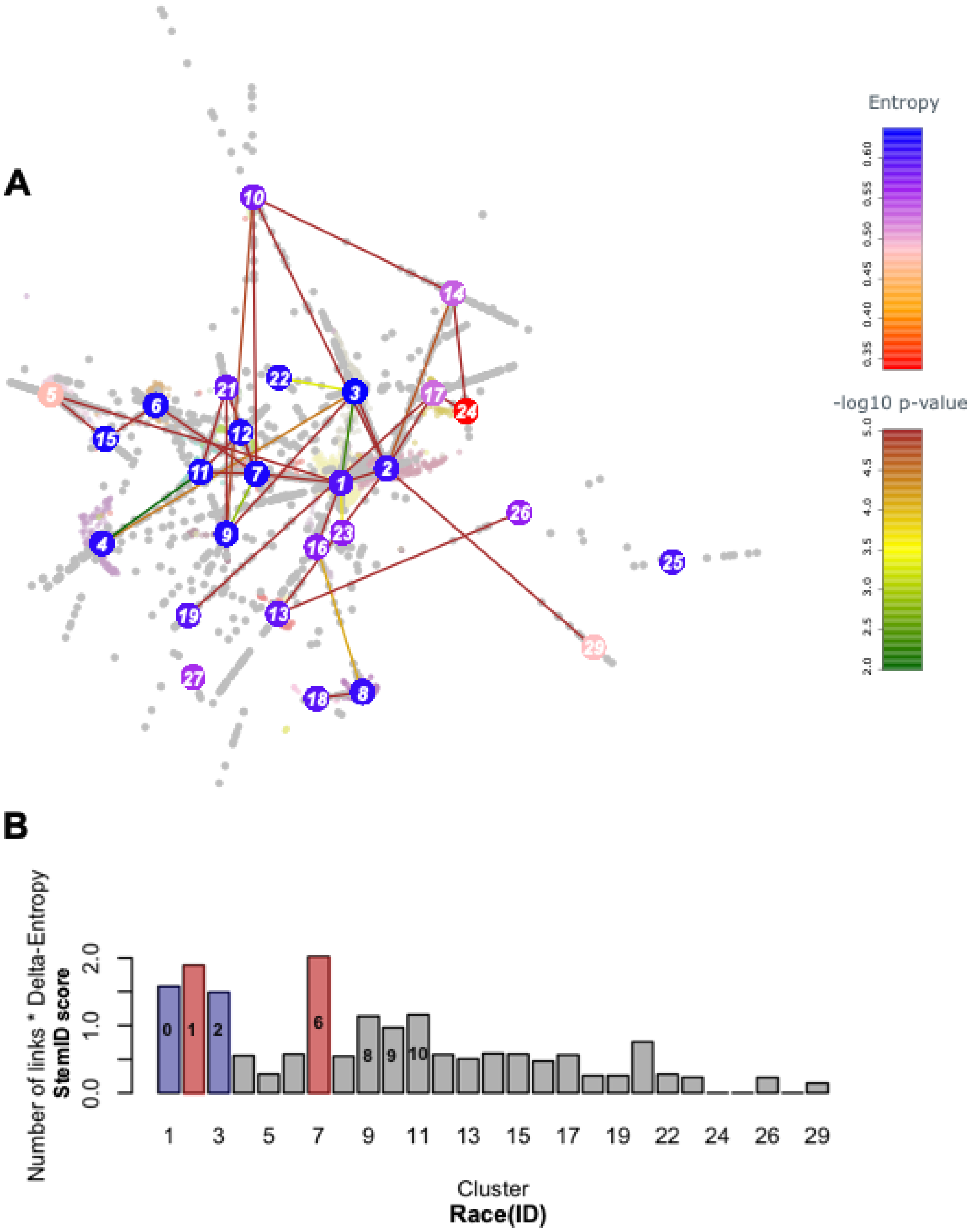

Fig. S21. StemID2 analysis identifies Seurat cluster 6 (RaceID cluster 7) as a putative progenitor population. A. Lineage graph generated by StemID2 using Seurat clustering (renumbered internally by RaceID). Node color reflects entropy; edge thickness corresponds to transcriptional similarity (link score), edge color depicts significance. RaceID cluster 7 (corresponding to Seurat cluster 6) is centrally positioned with multiple outgoing edges to clusters 1, 2, 6, 9, 11, and 12. B. Support for progenitor status. StemID score across clusters. RaceID cluster 7 (Seurat cluster 6) and 2 (Seurat cluster 1) show the highest StemID scores consistent with stem/progenitor identity. Clusters 1 and 2 (Seurat clusters 0 and 3) and 9-11 (Seurat clusters 8-10) also have high scores, consistent with transitional or multipotent states.

Table S1. Top DEG for each Seurat Cluster, includes genome ID, stringtie_ID and closest vertebrate orthologue.

Available for download at
https://journals.biologists.com/dev/article-lookup/doi/10.1242/dev.204265#supplementary-data

Table S2. Results from the GO and pathway analysis with Metascape.

Available for download at
https://journals.biologists.com/dev/article-lookup/doi/10.1242/dev.204265#supplementary-data

Table S3. Genes of interest for endostyle, stem and pluripotency factors and immune-related genes.

Available for download at
https://journals.biologists.com/dev/article-lookup/doi/10.1242/dev.204265#supplementary-data

Table S4. CellRank driver gene analysis for terminal clusters.

Available for download at
https://journals.biologists.com/dev/article-lookup/doi/10.1242/dev.204265#supplementary-data
